# Retraction: DNA-PK_CS_ binding to p53 on the *p21^WAF1/CIP1^* promoter blocks transcription resulting in cell death

**DOI:** 10.18632/oncotarget.27213

**Published:** 2019-09-17

**Authors:** Richard Hill, Patricia A. Madureira, David. M. Waisman, Patrick W.K. Lee

**Affiliations:** ^1^ Department of Microbiology & Immunology, Dalhousie University, Halifax, Nova Scotia, Canada; ^2^ Department of Biochemistry and Molecular Biology, Dalhousie University, Halifax, Nova Scotia, Canada; ^3^ Department of Pathology, Dalhousie University, Halifax, Nova Scotia, Canada

**This article has been retracted:** Due to numerous examples of splicing and transposition of data into figures, as well as other errors, the authors believe that retraction of this paper is the only responsible and logical course of action. As a result, all authors have agreed to the retraction of this paper from Oncotarget.

Original article: Oncotarget. 2011; 2:1094–1108. 1094-1108. https://doi.org/10.18632/oncotarget.378

